# Phenotypic upregulation of hexocylceramides and ether‐linked phosphocholines as markers of human extreme longevity

**DOI:** 10.1111/acel.14429

**Published:** 2024-12-05

**Authors:** Anna Fernàndez‐Bernal, Joaquim Sol, José Daniel Galo‐Licona, Natàlia Mota‐Martorell, Cristina Mas‐Bargues, Ángel Belenguer‐Varea, Èlia Obis, José Viña, Consuelo Borrás, Mariona Jové, Reinald Pamplona

**Affiliations:** ^1^ Department of Experimental Medicine University of Lleida‐Lleida Biomedical Research Institute (UdL‐IRBLleida) Lleida Spain; ^2^ Catalan Health Institute (ICS), Lleida Research Support Unit (USR) Fundació Institut Universitari per a la Recerca en Atenció Primària de Salut Jordi Gol i Gurina (IDIAP JGol) Lleida Spain; ^3^ Freshage Research Group, Department of Physiology, Faculty of Medicine, Centro de Investigación Biomédica en Red Fragilidad y Envejecimiento Saludable‐Instituto de Salud Carlos III (CIBERFES‐ISCIII) Institute of Health Research‐INCLIVA, University of Valencia València Spain; ^4^ Division of Geriatrics, Hospital Universitario de La Ribera (Alzira, Valencia, Spain), School of Doctorate Universidad Católica de Valencia Valencia Spain

**Keywords:** centenarians, cententarians' offspring, ether lipids, extreme longevity, hexocylceramides, lipidomics

## Abstract

Centenarians and their relatives possess a notable survival advantage, with higher longevity and reduced susceptibility to major age‐related diseases. To date, characteristic omics profiles of centenarians have been described, demonstrating that these individuals with exceptional longevity regulate their metabolism to adapt and incorporate more resilient biomolecules into their cells. Among these adaptations, the lipidomic profile stands out. However, it has not yet been determined whether this lipidomic profile is specific to centenarians or is the consequence of extreme longevity genetics and is also present in centenarians' offspring. This distinction is crucial for defining potential therapeutic targets that could help delay the aging process and associated pathologies. We applied mass‐spectrometry‐based techniques to quantify 569 lipid species in plasma samples from 39 centenarians, 63 centenarians' offspring, and 69 noncentenarians' offspring without familial connections. Based on this profile, we calculated different indexes to characterize the functional and structural properties of plasma lipidome. Our findings demonstrate that extreme longevity genetics (centenarians and centenarians' offspring) determines a specific lipidomic signature characterized by (i) an enrichment of hexosylceramides, (ii) a decrease of specific species of ceramides and sulfatides, (iii) a global increase of ether‐PC and ether‐LPC, and (iv) changes in the fluidity and diversity of specific lipid classes. We point out the conversion of ceramides to hexosylceramides and the maintenance of the levels of the ether‐linked PC as a phenotypic trait to guarantee extreme longevity. We propose that this molecular signature is the result of an intrinsic adaptive program that preserves protective mechanisms and cellular identity.

## Graphical abstract

Mass spectrometry analysis of 569 lipid species showed that ceramide‐to‐hexosylceramide conversion and the preservation of ether‐linked PC levels are key phenotypic traits ensuring extreme longevity. This lipidomic signature is shaped by an intrinsic adaptive program that maintains protective mechanisms and cellular identity.

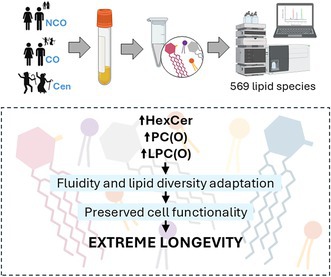

**Abbreviations**
ACL: Acyl Chain Length
Cer: Ceramides
CO: Centenarians’ Offspring
DBI: Double Bond Index
DG: Diacylglycerols
DvI: Diversity Index
FA: Fatty Acids
FlI: Fluidity Index
HDL: High‐Density Lipoprotein
Hex2Cer: Hexosyl2ceramides
Hex3Cer: Hexosyl3ceramides
HexCer: Hexosylceramides
HPLC: High‐Performance Liquid Chromatography
ISTD: Internal Standard
LC‐MS: Liquid Chromatography‐Mass Spectrometry
LDL: Low‐Density Lipoprotein
LPC: Lysophosphatidylcholine
LPC(O): Ether‐linked Lysophosphatidylcholine
NCO: Non‐Centenarians’ Offspring
PC: Phosphatidylcholine
PC(O): Ether‐linked Phosphatidylcholine
PE: Phosphatidylethanolamine
SAT: Saturated Species Ratio
TG: Triglycerides
VLDL: Very Low‐Density Lipoprotein

## 1 INTRODUCTION

Although life expectancy has been increasing over the past decades, only a few remarkable individuals reach high longevities in good health conditions (van den Berg, 2020). Among them, centenarians are considered a human model of healthy and successful aging (Francescshi et al., 2017; Perls et al., 2002) as well as of extreme longevity given their proximity to the maximum longevity of the human species, which is estimated to be between 115 and 120 years (Marck et al., 2017; Vijg & Le Bourg, 2017). Centenarians reach very advanced ages with highly preserved cognitive function and few comorbidities (Cruces‐Salguero et al., 2023; Francescshi et al., 2017), making the study of these individuals crucial to uncovering the biological mechanisms that lead to healthy aging and extreme longevity. In this regard, multiple studies have shown that the blood composition of centenarians differs from that of octogenarians and is more like that of younger adults, suggesting a characteristic lipidomic profile. This specific lipidome ensures a good remodeling of membrane lipids and confers greater resistance to oxidation and a better antioxidant defense (Collino et al., 2013; Jové et al., 2017; Montoliu et al., 2014; Mota‐Martorell et al., 2021; Pradas et al., 2019, 2022). However, a causal relationship between the described profiles and becoming a centenarian is yet to be demonstrated.

Epidemiological studies suggest that human longevity clusters in specific families (Ash et al., 2015). These findings indicate that relatives (including parents, siblings, and offspring) of long‐lived individuals have inherited, at least partially, the extreme longevity genetics and possess a notable survival advantage, with a higher likelihood of longevity, and a reduced susceptibility to major age‐related diseases (Adams et al., 2008; Atzmon et al., 2004; Barzilai et al., 2003; Bucci et al., 2016; Ikeda et al., 2006; Karasik et al., 2004; Perls et al., 2007; Schoenmaker et al., 2006; Terry et al., 2003; Terry, Wilcox, McCormick, Pennington, et al., 2004; Terry, Wilcox, McCormick & Perls et al., 2004; Westendorp et al., 2009; Willcox et al., 2006). Consequently, relatives of centenarians, particularly offspring, provide a valuable model for investigating the biological and genetic factors contributing to human healthy aging and extreme longevity.

The findings from various studies indicate a correlation between maximum longevity and lipid composition and, specifically, an increased resistance to lipid oxidation (Hulbert et al., 2007; Naudí et al., 2013; Pamplona, 2008). Supporting this hypothesis, a phylogenomic analysis revealed heightened selective pressure on genes involved in lipid metabolism in long‐lived species (Jobson et al., 2010). Expanding on these findings, different lipidomic studies have confirmed the species‐specific nature of the lipidome, which is also associated with species' longevity (Bozek et al., 2017; Jové et al., 2013). Additionally, certain studies have identified favorable lipid profiles in the offspring of centenarians, indicating genotypic adaptations in lipid metabolism linked to human longevity (Barzilai et al., 2001; Vaarhorst et al., 2011). Taken together, these observations highlight lipids as a critical target for investigating the molecular adaptive mechanisms underlying differences in human longevity.

Based on these premises, the main goal of the present study is to define the lipidomic signature of extreme longevity analyzing individuals who reach 100 years old and offsprings of +100 years old individuals. It is known that healthy aging and longevity depend on genetics (about 18–30%) but also environmental and lifestyle determinants (Graham Ruby et al., 2018). In the present study, the offsprings of centenarians belong to independent families from the individuals of centenarian group. This is because the intention is to comprehensively describe the lipidomic footprint of extreme longevity, rather than focusing solely on the longevity of a few families.

## 2 METHODS

### 2.1 Participants

The study includes 39 centenarians, 63 centenarian offspring (CO), and 69 noncentenarian offspring (NCO) without familial connections living within the area of La Ribera (11th Health Department of the Valencian Community, Valencia, Spain), as previously described (Inglés et al., 2022). Prior to participation, all individuals or their relatives were informed on the study's objectives, scope, and associated risks. Written consent was obtained from all participants. Inclusion criteria were delineated as follows: (i) for centenarians, an age of 97 or older at the beginning of the study; (ii) for CO, individuals aged 65–80 dwelling in the community with at least one parent having lived to or beyond 97 years; and (iii) for control NCO, individuals matched with CO in terms of age (±5 years), gender, birthplace, and residence, but with parents who did not live beyond 90 years. Additionally, all participants had to be permanent residents (residing more than 6 months annually) in the health jurisdiction of La Ribera (Valencia, Spain) and registered in the local population database. Exclusion criteria for all groups included terminal illness or a life expectancy of less than 6 months due to any cause. All procedures adhered to the principles of the Declaration of Helsinki by the World Medical Association and complied with national and international standards. The study protocol was endorsed by the Committee for Ethics in Clinical Research at Hospital de la Ribera, Alzira, Spain.

### 2.2 Sampling

Blood samples were drawn from the antecubital vein in VACUTAINER CPT tubes (BD, Franklin Lakes, NJ) containing sodium heparin as an anticoagulant, to obtain plasma samples. Within 0.5 h of collection, blood was processed at the collection site according to the manufacturer's instructions by centrifugation at 3000*g* at room temperature for 15 min. Plasma samples were collected and frozen at −80°C for subsequent lipidomic analysis. Laboratory personnel were all blinded to the sample's identity.

### 2.3 Biochemical assays

Glucose, total cholesterol, HDL cholesterol, and total triglycerides were determined by standard biochemical assays. The concentration of LDL cholesterol was calculated by using the Friedewald equation: LDL = total cholesterol − HDL − (total triglycerides/5).

### 2.4 Targeted lipidomic analysis

Lipidome extraction was performed as described previously (Alshehry et al., 2015). In brief, 10 μL of plasma was mixed with 100 μL of butanol: methanol (1:1) with 10 mM ammonium formate which contained a mixture of internal standards (Table S1). Samples were vortexed and set in a sonicator bath maintained at room temperature for 1 h. Samples were then centrifuged (16000g, RT, 10min) before transferring them into sample vials with glass inserts for analysis.

Lipid extracts were analyzed by LC–MS on an Agilent 6495 LC/QC mass spectrometer with an Agilent 1290 series HPLC system and a ZORBAX eclipse plus C18 column (2.1 × 100 mm 1.8 mm, Agilent) with the thermostat set at 60°C. Mass spectrometry analysis was performed in positive and negative ion mode with dynamic scheduled multiple reaction monitoring (MRM).

The solvent system consisted of solvent A: 50% H_2_O/30% acetonitrile/20% isopropanol (v/v/v) containing 10 mM ammonium formate and solvent B: 1% H_2_O/9% acetonitrile/90% isopropanol (v/v/v) containing 10 mM ammonium formate. The gradient was as follows; starting with a flow rate of 0.4 mL/min at 10% B and increasing to 45% B over 2.7 min, then to 53% over 0.1 min, to 65% over 6.2 min, to 89% over 0.1 min, to 92% over 1.9 min, and finally to 100% over 0.1 min. The solvent was then held at 100% B for 2.3 min (a total of 13.4 min). Equilibration was as follows, solvent was decreased from 100% B to 10% B over 0.1 min and held for an additional 0.9 min. The flow rate was then switched to 0.6 mL/min for 1 min before returning to 0.4 mL/min over 0.1 min. Solvent B was held at 10% B for a further 0.9 min at 0.4 mL/min for a total cycle time of 16.5 min.

For quantification, chromatographic peaks were assigned to each lipid based on specified transitions (precursor>product ion) and retention time. Peaks were integrated using the Mass Hunter Agilent Mass Hunter Quantitative Analysis 10.1 software (Agilent Technologies). Quantification was achieved by using the ratio between each compound peak and the corresponding internal standard. The whole dataset with the lipid intensities was introduced to the SERRF online platform (Fan et al., 2019), and triglycerides and cholesterol esters normalized signals were divided by the respective ISTD median signals to calculate the concentrations.

To further characterize the functional and structural properties of plasma lipidome, different indexes were calculated:

*Double Bond Index* (*DBI*): Sum, for each lipid species in a category (i.e., class/subclass/other classification): (species concentration × number of double bonds/number of acyl chains)/total concentration of lipids in the category.

*Acyl Chain Length* (*ACL*): Sum, for each lipid species in a category (i.e., class/subclass/other classification): (species concentration × number of carbons/number of acyl chains)/total concentration of lipids in the category.

*Saturated species ratio* (*SAT*): Concentration of saturated species in the category/total concentration of lipids in the category.

*Fluidity Index* (*FlI*) (Osetrova et al., 2024): Sum, for each lipid species in a category (i.e., class/subclass/other classification): (species concentration × fluidity contribution)/total concentration of lipids in the category.

Fluidity contribution = average of chain length contribution and double bond contribution, where.

Chain length contribution = 1−(single chain length of the lipid species‐minimum single chain length in the category)/range of single chain lengths in the category.

Double bond contribution = (single chain number of double bonds of the lipid species‐minimum single chain number of double bonds in the category)/range of single chain number of double bonds in the category.

*Diversity Index* (*DvI*) (Simpson, 1949): Simpson's index of diversity = 1−*D*. The index follows the rationale from the diversity index calculated in ecology, which indicates the probability that two random individuals picked from an ecosystem belong to a different species, where *D* is the number of possible pairs from the same species that can be drawn from the sample/total number of possible pairs.

$$D=\frac{\sum n\left(n-1\right)}{N\left(N-1\right)}$$

*n* = number of individuals from a species.

*N* = total number of individuals in the ecosystem.

We applied this concept to the lipid concentrations to calculate the diversity of plasma lipid species within the same lipid class or within the lipidome to know whether it is related to successful aging or not. Higher values indicate higher diversity of lipids and lower values indicate lower diversity of lipids. We used the alpha.div function from the asbio *R* package. We calculated the index based on concentrations rather than the number of lipids because most physicochemical properties are related to lipid content.

### 2.5 Statistical analysis

Anthropometric and clinical variables were compared using a Kruskal–Wallis test or an ANOVA depending on their distribution based on a Shapiro–Wilk test.

Lipid concentrations were standardized by calculating their z‐scores. Multivariate analyses (Principal Component Analysis and heatmaps) were performed using Metaboanalyst (Chong et al., 2019). The lipid concentrations of centenarians, CO, and NCO were compared using Kruskal–Wallis tests and the corresponding Dunn's post‐hoc test. Overrepresentation of lipids in each class was calculated using a hypergeometric test. Statistical significance was set at false discovery rate adjusted *p*‐values <0.05.

## 3 RESULTS

### 3.1 Sample characteristics

The main anthropometric and clinical variables of the study's population are shown in Table 1. There were no statistically significant differences in age, height, and general lipid profile between CO and NCO, but CO showed lower levels of weight, BMI, and glucose. Both CO and NCO were younger and presented higher height than centenarians. Centenarians and CO showed lower plasma glucose levels than NCO.

**TABLE 1:** Baseline comparisons of individuals' anthropometric and biochemical parameters.

	NCO (*N* = 69)	CO (*N* = 63)	Cent (*N* = 39)	*p*.overall	*p*.Cent vs. CO	*p*.Cent vs. NCO	*p*.CO vs. NCO
Female, *n* (%)	42 (60.9%)	36 (57.1%)	30 (76.9%)	0.116	0.205	0.205	0.797
Age (y), median [Q1;Q3]	67.0 [66.0;72.0]	70.0 [66.5;73.0]	99.0 [98.0;100]	**<0.001**	**<0.001**	**<0.001**	0.589
Weight (kg), mean (SD)	78.2 (13.3)	71.1 (13.9)	61.9 (15.7)	**<0.001**	**0.006**	**<0.001**	**0.013**
Height (m), mean (SD)	1.61 (0.08)	1.60 (0.09)	1.52 (0.09)	**<0.001**	**<0.001**	**<0.001**	0.784
BMI (kg/m^2^), median [Q1;Q3]	30.1 [26.8;33.3]	28.1 [25.2;29.4]	25.2 [21.8;30.4]	**0.001**	0.108	**0.004**	**0.006**
Glucose (mg/dL), median [Q1;Q3]	101 [93.0;119]	93.0 [87.0;101]	87.0 [81.0;93.0]	**<0.001**	**0.004**	**<0.001**	**0.004**
Total Cholesterol (mg/dL), median [Q1;Q3]	188 [168;217]	202 [176;230]	179 [164;194]	**0.017**	**0.026**	0.233	0.066
HDL cholesterol (mg/dL), median [Q1;Q3]	54.0 [46.0;63.0]	58.0 [47.5;69.5]	50.0 [40.5;62.0]	0.065	0.072	0.204	0.204
LDL cholesterol (mg/dL), median [Q1;Q3]	110 [91.0;131]	118 [97.5;148]	107 [88.0;130]	0.245	0.248	0.701	0.248
VLDL cholesterol (mg/dL), median [Q1;Q3]	23.0 [17.0;30.0]	23.0 [16.5;30.0]	21.0 [16.0;27.0]	0.497	0.513	0.513	0.804
Total Triglycerides (mg/dL), median [Q1;Q3]	116 [83.0;152]	113 [82.5;150]	107 [78.0;134]	0.511	0.538	0.538	0.783
ApoB (mg/dL), median [Q1;Q3]	97.7 (21.6)	102 (18.4)	94.4 (21.6)	0.188	0.176	0.704	0.469

*Note*: Bold indicates *p* < 0.05.

### 3.2 Global lipidomic profile

**FIGURE 1:**
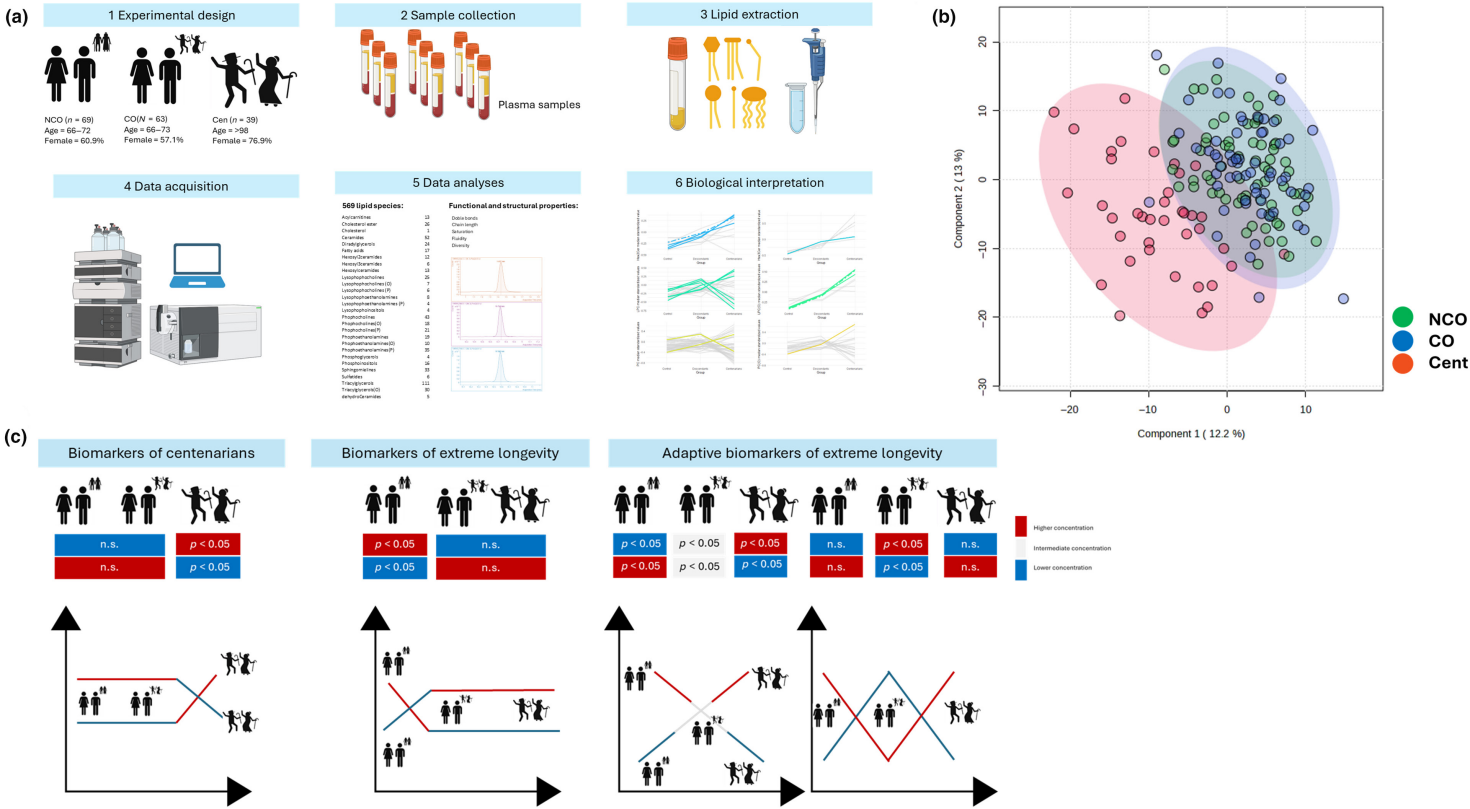
(a) Lipidomic workflow used for the study. (b) Principal Component Analysis using all the lipid species detected. (c) Biological interpretation: Biomarkers of centenarians: Those lipids that are significantly different between centenarian and noncentenarian (centenarians' offsprings and noncentenarians' offspring) individuals. Biomarkers of extreme longevity: Those lipids that are common in individuals with longevous genetic background (centenarians and centenarians offsprings) during life. Adaptive biomarkers of extreme longevity: (i) those biomarkers that are statistically different between control and centenarians' offsprings; and (ii) those biomarkers that are different between three groups, and the concentrations of these species in centenarians' offsprings are intermediate between control and centenarians. CO, Centenarians' offspring, Cent, Centenarians; NCO, Noncentenarians' offspring.

The lipidomic workflow applied in this study is shown in Figure 1a. A targeted lipidomic approach including 569 lipid species belonging to all the lipid classes was applied to characterize the plasma lipidomic profile. Based on this characterization, different indexes (saturation (SAT), Average Chain Length (ACL), Double Bond Index (DBI), Fluidity Index (FlI), and Diversity Index (DvI)) were calculated to define the functional and structural properties of the plasma lipidome. The mean and median concentration of each lipid is shown in Supplementary Dataset (Data S1).

**FIGURE 2:**
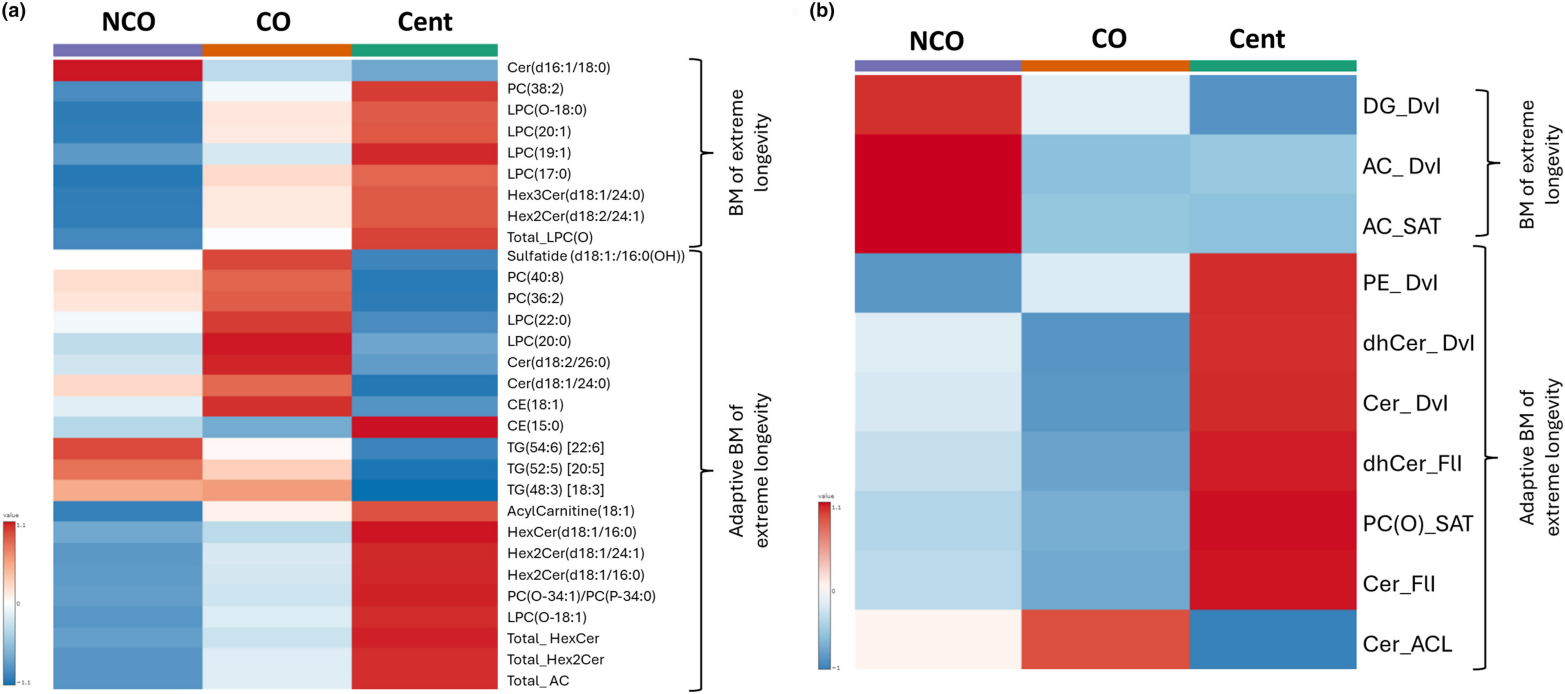
Heatmap representation of the lipid species that defines the lipidomic fingerprint of extreme longevity (a) and the functional and structural properties of the lipidome (b). FlI is calculated according to chain length and double bonds of their fatty acids. DvI represents the probability that two lipids randomly selected from a sample will belong to different species. Higher diversity values indicate higher diversity of lipids within the specified lipid class. AC, Aclycarnitines; ACL, Average Chain Length; CE, Cholesterol esters; Cent, Centenarians; Cer, Ceramides; CO, Centenarians' offspring; DBI, Double Bond Index; dhCer, Dihydroceramide; DvI, Diversity Index; FA, Fatty acids; FlI, Fluidity Index; Hex2Cer, Hexosyl2ceramides; Hex3Cer, Hexosyl3ceramides; HexCer, Hexosylceramides; LPC(O), Lysophosphatidylcholine(O); LPC(P), Lysophosphatidylcholine(P); LPC, Lysophosphatidylcholine; NCO, Noncentenarians' offspring; PC(O), Phosphatidylcholine(O); PC(P), Phosphatidylcholine(P); PC, Phosphatidylcholine; SAT, Saturation; TG, Triglycerides; Total_AC, Sum of the concentration of all AC species; Total_Hex2Cer, Sum of the concentration of all Hex2Cer species; Total_HexCer, Sum of the concentration of all HexCer species; Total_LPC(O), Sum of the concentration of all LPC(O) species.

Unsupervised multivariate statistics (Principal Component Analysis) (Figure 1b) of the global lipidome revealed that centenarians possess a distinct plasma lipidomic profile, which is clearly separated and differentiated from the lipidomes of CO and NCO. When univariate statistics were applied (Kruskal–Wallis test) 12 biomarkers of extreme longevity and 28 adaptive biomarkers of extreme longevity arose (Table 2). In addition to this, 215 lipid species and indexes arose as biomarkers of centenarians (Supplementary Dataset [Data S1]). The biological interpretation of the statistically significant biomarkers is represented and explained in Figure 2c.

**TABLE 2:** Biomarkers of extreme longevity.

Feature	FDR p‐value	Post‐hoc (Dunn's test)	*p* (cent‐NCO)	*p* (cent‐CO)	*p* (NCO‐CO)	NCO (median [Q1;Q3]) pmol/μL	CO (median [Q1;Q3]) pmol/ μL	Cent (median [Q1;Q3]) pmol/μL
Biomarkers of extreme longevity								
AC_DvI	3.91E‐02	Cent–NCO, NCO–CO	1.72E‐02	4.92E‐01	1.34E‐02	0.84 [0.83;0.85]	0.84 [0.82;0.85]	0.84 [0.82;0.84]
AC_SAT	7.65E‐03	Cent–NCO, NCO–CO	2.29E‐03	3.54E‐01	4.14E‐03	0.32 [0.30;0.35]	0.30 [0.27;0.34]	0.29 [0.28;0.32]
Cer(d16:1/18:0)	4.20E‐02	Cent–NCO, NCO–CO	1.99E‐02	3.41E‐01	1.26E‐02	0.04 [0.03;0.05]	0.03 [0.03;0.04]	0.03 [0.02;0.04]
DG_DvI	4.97E‐03	Cent–NCO, NCO–CO	5.40E‐04	6.32E‐02	1.43E‐02	0.83 [0.82;0.85]	0.82 [0.81;0.84]	0.81 [0.80;0.82]
Hex2Cer(d18:2/24:1)	1.38E‐02	Cent–NCO, NCO–CO	2.78E‐03	1.51E‐01	1.23E‐02	0.15 [0.11;0.18]	0.16 [0.13;0.22]	0.19 [0.13;0.24]
Hex3Cer(d18:1/24:0)	7.00E‐03	Cent–NCO, NCO–CO	7.58E‐04	5.84E‐02	2.12E‐02	0.26 [0.22;0.32]	0.31 [0.23;0.41]	0.33 [0.29;0.42]
LPC(17:0)	3.32E‐02	Cent–NCO, NCO–CO	1.07E‐02	2.46E‐01	1.55E‐02	0.81 [0.63;1.01]	0.88 [0.73;1.10]	1.04 [0.71;1.24]
LPC(19:1)	8.48E‐03	Cent–NCO, NCO–CO	1.07E‐03	7.31E‐02	1.99E‐02	0.03 [0.02;0.04]	0.03 [0.03;0.04]	0.04 [0.03;0.05]
LPC(20:1)	1.46E‐02	Cent–NCO, NCO–CO	7.30E‐03	3.35E‐01	4.30E‐03	0.11 [0.10;0.15]	0.14 [0.12;0.18]	0.15 [0.10;0.20]
LPC(O‐18:0)	2.50E‐02	Cent–NCO, NCO–CO	7.08E‐03	2.19E‐01	1.36E‐02	0.62 [0.48;0.79]	0.70 [0.58;0.83]	0.85 [0.55;1.01]
PC(38:2)	5.60E‐03	Cent–NCO, NCO–CO	7.01E‐04	8.19E‐02	1.15E‐02	18.3 [15.2;22.9]	21.0 [18.3;24.1]	22.9 [18.3;29.9]
Total_LPC(O)	1.56E‐02	Cent–NCO, NCO–CO	2.30E‐03	9.31E‐02	2.53E‐02	1.95 [1.64;2.36]	2.11 [1.87;2.59]	2.51 [1.83;2.90]
Adaptive biomarkers of extreme longevity								
AcylCarnitine(18:1)	3.59E‐04	Cent–NCO, Cent–CO, NCO–CO	2.00E‐05	2.81E‐02	3.73E‐03	0.96 [0.72;1.11]	1.08 [0.92;1.33]	1.27 [1.06;1.49]
CE(15:0)	2.54E‐06	Cent–NCO, Cent–CO, NCO–CO	2.71E‐05	2.19E‐08	3.20E‐02	6.16 [4.62;7.69]	5.06 [3.64;6.37]	8.81 [6.46;11.2]
CE(18:1)	3.32E‐02	Cent–CO, NCO–CO	1.36E‐01	6.55E‐03	2.89E‐02	539 [467;612]	589 [516;650]	510 [447;566]
Cer(d18:1/24:0)	8.24E‐06	Cent–NCO, Cent–CO, NCO–CO	5.71E‐05	9.70E‐08	4.02E‐02	7.45 [6.22;8.81]	8.40 [6.54;9.29]	5.81 [4.76;7.46]
Cer(d18:2/26:0)	1.14E‐02	Cent–CO, NCO–CO	1.58E‐01	2.44E‐03	8.57E‐03	0.02 [0.02;0.03]	0.03 [0.02;0.03]	0.02 [0.02;0.02]
Cer_ACL	1.53E‐05	Cent–NCO, Cent–CO, NCO–CO	3.96E‐04	1.60E‐07	1.36E‐02	20.5 [20.5;20.6]	20.6 [20.5;20.6]	20.5 [20.5;20.5]
Cer_DvI	3.82E‐06	Cent–NCO, Cent–CO, NCO–CO	1.32E‐04	3.20E‐08	1.33E‐02	0.85 [0.83;0.86]	0.84 [0.82;0.85]	0.86 [0.85;0.86]
Cer_FlI	2.59E‐10	Cent–NCO, Cent–CO, NCO–CO	2.64E‐09	4.72E‐13	4.49E‐02	0.20 [0.20;0.20]	0.20 [0.20;0.20]	0.20 [0.20;0.20]
dhCer_DvI	5.50E‐07	Cent–NCO, Cent–CO, NCO–CO	7.81E‐06	2.84E‐09	2.68E‐02	0.73 [0.71;0.73]	0.72 [0.71;0.73]	0.74 [0.73;0.74]
dhCer_FlI	4.16E‐07	Cent–NCO, Cent–CO, NCO–CO	4.15E‐06	2.03E‐09	3.32E‐02	0.17 [0.16;0.17]	0.16 [0.16;0.17]	0.17 [0.17;0.17]
HexCer(d18:1/16:0)	2.51E‐06	Cent–NCO, Cent–CO, NCO–CO	1.62E‐08	9.82E‐05	1.60E‐02	1.58 [1.34;1.95]	1.82 [1.46;2.06]	2.45 [1.82;3.05]
Hex2Cer(d18:1/16:0)	4.30E‐03	Cent–NCO, Cent–CO, NCO–CO	3.07E‐04	2.94E‐02	2.96E‐02	3.04 [2.51;3.61]	3.39 [2.83;3.89]	3.65 [3.01;4.85]
Hex2Cer(d18:1/24:1)	3.34E‐05	Cent–NCO, Cent–CO, NCO–CO	6.37E‐07	3.74E‐03	4.79E‐03	0.90 [0.70;1.06]	1.02 [0.83;1.24]	1.22 [0.96;1.49]
LPC(20:0)	3.41E‐02	Cent–CO, NCO–CO	2.00E‐01	8.99E‐03	1.86E‐02	0.07 [0.06;0.09]	0.08 [0.07;0.10]	0.07 [0.04;0.09]
LPC(22:0)	3.97E‐03	Cent–NCO, Cent–CO, NCO–CO	3.43E‐02	3.12E‐04	1.90E‐02	0.02 [0.02;0.02]	0.02 [0.02;0.03]	0.02 [0.01;0.02]
LPC(O‐18:1)	1.12E‐03	Cent–NCO, Cent–CO, NCO–CO	5.07E‐05	1.64E‐02	1.71E‐02	0.52 [0.43;0.61]	0.56 [0.48;0.71]	0.67 [0.52;0.85]
PC(36:2)	1.07E‐03	Cent–NCO, Cent–CO, NCO–CO	6.21E‐03	4.37E‐05	3.30E‐02	262 [222;303]	277 [250;318]	231 [166;263]
PC(40:8)	2.21E‐04	Cent–NCO, Cent–CO, NCO–CO	9.66E‐04	6.47E‐06	4.90E‐02	0.62 [0.52;0.77]	0.71 [0.56;0.84]	0.48 [0.38;0.60]
PC(O)_SAT	6.51E‐06	Cent–NCO, Cent–CO, NCO–CO	6.94E‐05	6.82E‐08	3.03E‐02	0.02 [0.01;0.02]	0.01 [0.01;0.02]	0.02 [0.02;0.02]
PC(P‐34:0)/PC(O‐34:1)	1.13E‐04	Cent–NCO, Cent–CO, NCO–CO	2.37E‐06	1.78E‐03	2.26E‐02	7.11 [5.95;8.44]	7.94 [6.38;9.29]	9.69 [7.24;11.2]
PE_DvI	7.90E‐04	Cent–NCO, Cent–CO, NCO–CO	2.97E‐05	8.89E‐03	2.29E‐02	0.86 [0.85;0.86]	0.86 [0.86;0.87]	0.87 [0.86;0.87]
Sulfatide (d18:1:/16:0(OH))	7.54E‐03	Cent–NCO, Cent–CO, NCO–CO	3.59E‐02	6.92E‐04	3.27E‐02	0.12 [0.09;0.14]	0.13 [0.10;0.17]	0.10 [0.08;0.12]
TG(48:3) [18:3]	8.07E‐03	Cent–NCO, Cent–CO, NCO–CO	6.96E‐04	3.73E‐02	3.93E‐02	0.04 [0.03;0.06]	0.03 [0.02;0.05]	0.03 [0.01;0.03]
TG(52:5) [20:5]	1.12E‐04	Cent–NCO, Cent–CO, NCO–CO	2.33E‐06	3.05E‐03	1.38E‐02	0.03 [0.02;0.04]	0.02 [0.01;0.04]	0.01 [0.01;0.02]
TG(54:6) [22:6]	8.55E‐05	Cent–NCO, Cent–CO, NCO–CO	1.84E‐06	6.30E‐03	5.63E‐03	0.16 [0.11;0.22]	0.11 [0.08;0.19]	0.09 [0.06;0.11]
Total_AC	3.48E‐03	Cent–NCO, Cent–CO, NCO–CO	2.28E‐04	2.92E‐02	2.44E‐02	3.20 [2.41;3.76]	3.62 [2.79;4.31]	3.97 [3.48;4.92]
Total_Hex2Cer	3.69E‐03	Cent–NCO, Cent–CO, NCO–CO	2.52E‐04	3.34E‐02	2.24E‐02	5.74 [4.59;6.54]	6.11 [5.19;7.19]	6.75 [5.53;8.40]
Total_HexCer	2.32E‐04	Cent–NCO, Cent–CO, NCO–CO	6.50E‐06	2.58E‐03	2.89E‐02	12.7 [10.8;14.9]	14.0 [11.2;16.3]	16.8 [13.9;21.1]

*Note*: FlI is calculated according to chain length and double bonds of their fatty acids. DvI represents the probability that two lipids randomly selected from a sample will belong to different species. Higher diversity values indicate higher diversity of lipids within the specified lipid class.

Abbreviations: AC, aclycarnitines; ACL, Average Chain Length; CE, cholesterol esters; Cent, centenarians; Cer, Ceramides; CO, centenarians' offspring; DG, diacylglyceride; dhCer, Dihydroceramide; DvI, Diversity Index; FlI, Fluidity Index; Hex2Cer, hexosyl2ceramides; Hex3Cer, hexosyl3ceramides; HexCer, hexosylceramides; LPC(O), Lysophosphatidylcholine(O); LPC, Lysophosphatidylcholine; NCO, noncentenarians' offspring; PC(O), Phosphatidylcholine(O); PC, Phosphatidylcholine; PE, phosphatidylethanolamine; SAT, saturation; TG, triglycerides; Total_AC, sum of the concentration of all AC species; Total_Hex2Cer, sum of the concentration of all Hex2Cer species; Total_HexCer, sum of the concentration of all HexCer species; Total_LPC(O), sum of the concentration of all LPC(O) species.

### 3.3 Lipidome profile associated with extreme longevity

The lipid species that define the lipidomic fingerprint of extreme longevity are represented in Figure 2a. Among those biomarkers of extreme longevity, we found one ceramide (Cer(d16:1/18:0)) decreased in extreme longevity genotype (centenarians and CO); and one phosphocholine (PC(38:2)), one ether‐linked lysophosphatidylcholine (LPC(O‐18:0)), three lysophosphatidylcholines (LPC(20:1), LPC(19:1) and LPC(17:0)), one hexosyl3ceramide (Hex3Cer(d18:1/24:0)) and one hexosyl2ceramide (Hex2Cer(d18:2/24:1)) increased in extreme longevity genotype. Furthermore, the total levels of ether‐linked lysophosphatidylcholines were associated with extreme longevity.

Regarding adaptive biomarkers of extreme longevity, we found four different behaviors: (i) one sulfatide (sulfatide(d18:1/16:0(OH))), two phosphocholines (PC(40:8) and PC(36:0)), two lysophosphatidylcholines (LPC(22:0) and LPC(20:0)), two ceramides (Cer(d18:2/26:0) and Cer(d18:1/24:0)) and one cholesterol ester (CE(18:1)) decreased in NCO and centenarians when compared to CO; (ii) the cholesterol ester CE(15:0) showed the opposite direction, being increased in NCO and centenarians compared to CO; (iii) one acylcarnitine (AcylCar(18:1)), one hexosylceramide (HexCer(d18:1/16:0)), two hexosyl2ceramide (Hex2Cer(d18:1/16:0) and Hex2Cer(d18:1/24:1)), one ether‐linked phosphocholine (PC(O‐34:1/PC(P‐34:0))), one ether‐linked lysophosphatidylcholine (LPC(O‐18:1)) and the total levels of hexosylceramides, hexosyl2ceramides and acylcarnitines (Total_HexCer, Total_Hex2Cer, and Total_AC) showed lower levels in NCO, intermediate levels in CO, and higher levels in centenarians; and finally, (iv) the three triglycerides (TG(54:6), TG(52:5) and TG(48:3)) showed higher levels in NCO, intermediate levels in CO, and lower levels in centenarians.

**FIGURE 3:**
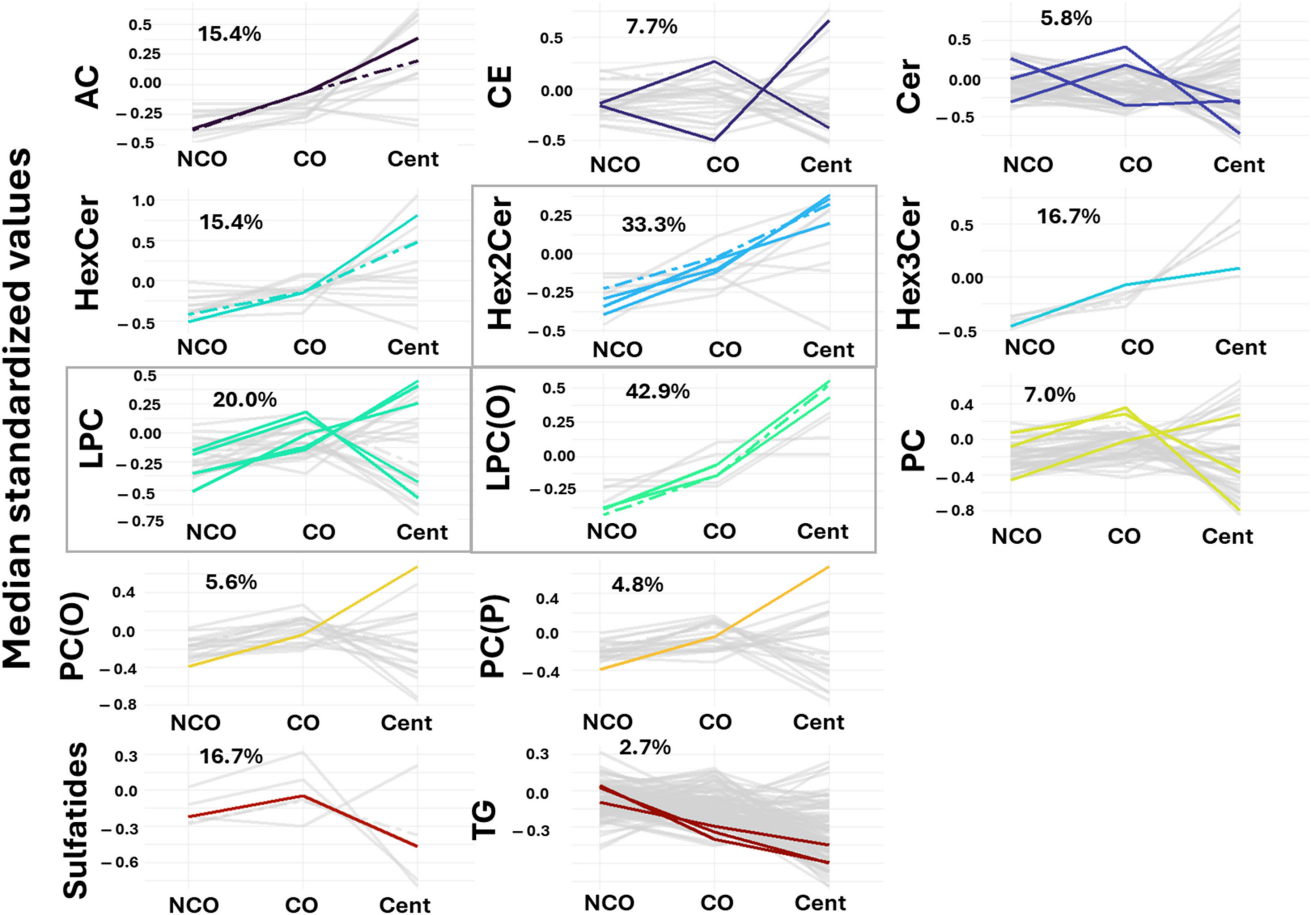
Lipid species significantly associated with extreme longevity genotype (centenarians and centenarians offsprings) grouped by class. Line chart representing all the lipid species of each class. Total concentration of each class is represented by dot‐dashed lines. Only statistically different species are colored. The percentage of lipid species affected by extreme longevity with respect to total species detected as indicated for each class. Overrepresentation of lipids in each class is calculated using a hypergeometric test and overrepresented classes are framed in gray (Hex2Cer, LPC, and LPC(O)). AC, Aclycarnitines; CE, Cholesterol esters; Cent, Centenarians; Cer, Ceramides; CO, Centenarians' offspring; FA, Fatty acids; Hex2Cer, Hexosyl2ceramides; Hex3Cer, Hexosyl3ceramides; HexCer, Hexosylceramides; LPC(O), Lysophosphatidylcholine(O); LPC(P), Lysophosphatidylcholine(P); LPC, Lysophosphatidylcholine; NCO, Noncentenarians' offspring; PC(O), Phosphatidylcholine(O); PC(P), Phosphatidylcholine(P); PC, Phosphatidylcholine; TG, Triglycerides.

To better characterize the differences in lipid profile associated with extreme longevity, lipid species associated with extreme longevity genotype (centenarians and CO) were grouped by class (Figure 3). Our results demonstrated that Hex2Cer, LPC, and ether‐linked LPC were overrepresented classes and, therefore, the most important classes defining extreme longevity. Specifically, 33.3% of these Hex2Cer detected were significantly associated with extreme longevity, 20% of LPCs, and 42.9% of ether‐linked LPCs.

### 3.4 Functional and structural properties of the lipidome

**FIGURE 4:**
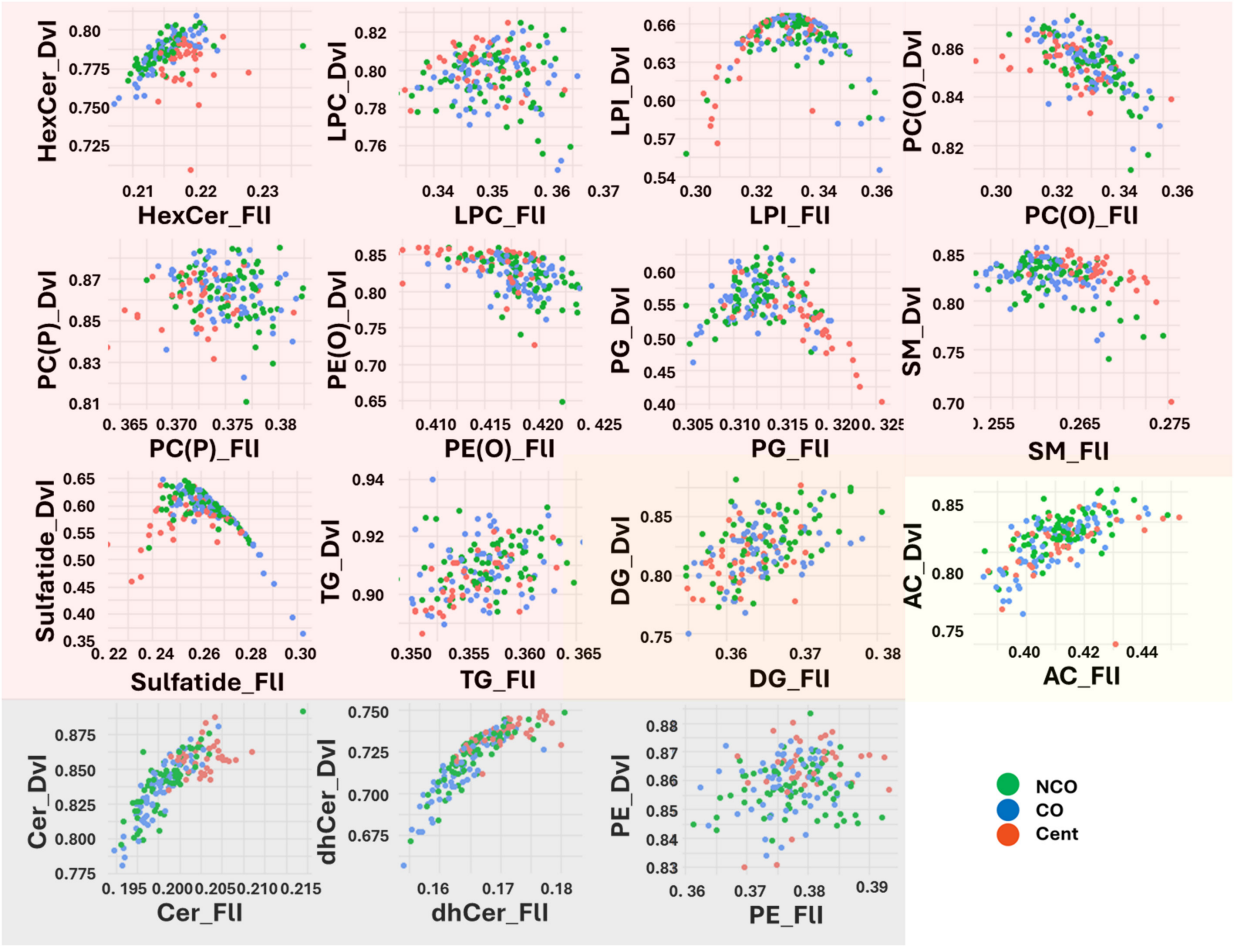
Functional properties of lipid species grouped by lipid class. Only the lipid classes that present statistically significant differences in Diversity Index (DvI) and/or Fluidity Index (FlI) between groups are selected. Biomarkers of centenarians are colored in red; biomarkers of extreme longevity in yellow and adaptive biomarkers of extreme longevity in gray. AC, Acylcarnitine; Cer, Ceramide; DG, Diglyceride; dhCer, Dihydroceramide; HexCer, HexosylCeramides; LPC, Lysophosphocholines; LPI, Lysophosphatidylinositol; PC(O), Phosphocholine(O); PC(P), Phosphocholine(P); PE(O), Phosphoethanolamine(O); PE, Phosphoethanolamine; PG, Phosphoglycerol; SM, Sphingomyeline; TG, Trygliceride. DvI represents the probability that two lipids randomly selected from a sample will belong to different species. Higher diversity values indicate higher diversity of lipids within the specified lipid class.

Lipid species composition determines functional and structural properties. First of all, the mean number of double bonds (DBI), the average chain length (ACL), and the saturated species ratio (SAT) for each lipid species in a category were calculated. After that, the Fluidity Index (FlI) was calculated according to ACL and DBI of their fatty acids. Finally, the Diversity Index (DvI), which represents the probability that two lipids randomly selected from a sample will belong to different species, was calculated. Higher diversity values of DvI indicate higher diversity of lipids within the specified lipid class. The functional properties of lipid species grouped by class are represented in Figure 4 (those that had statistically significant differences in DvI and/or FlI between groups) and in Figure S1 (those that are not considered biomarkers of centenarians, extreme longevity, and adaptive biomarkers of extreme longevity).

Among all the indexes calculated, three arose as extreme longevity biomarkers: DvI of diacylglycerols (DG), and DVI and FlI of acylcarnitines (Figure 2b). All three were decreased in extreme longevity genotypes (CO and centenarians). Furthermore, we found 7 adaptive biomarkers of extreme longevity. Specifically, we found three different behaviors: (i) the DvI of phosphatidylethanolamines (PE) showed lower levels in NCO, intermediate levels in CO, and higher levels in centenarians; (ii) the DvI and FlI of ceramides and dihydroceramides, and the SAT of ether‐linked PC showed intermediate levels in NCO, lower levels in CO, and higher levels in centenarians, and finally, (iii) ACL of ceramides showed intermediate levels in NCO, higher levels in CO, and lower levels in centenarians. All in all, the main lipid classes that present specific functional and structural properties in extreme longevity are DG, acylcarnitines, ceramides, dihydroceramides, ether‐linked PC, and PE.

## 4 DISCUSSION

Centenarians have been considered models of successful aging, and specific omic profiles for these exceptional individuals have been described, including genomics (Sebastiani et al., 1994), transcriptomics (Borras et al., 2016; Passtoors et al., 2013), metabolomics (Collino et al., 2013), lipidomics (Collino et al., 2013; Gonzalez‐Covarrubias et al., 2013; Jové et al., 2017; Pradas et al., 2019, 2022), metagenomics, and microbiomics (Biagi et al., 2017; Li et al., 2022). Although these individuals are a good model for studying extreme longevity, we do not know whether those omic profiles are specific for centenarians or are specific to an extreme longevity genotype (centenarians and relatives). In this work, we demonstrate that there is a specific lipidomic profile for extreme longevity, which is primarily characterized by a differential regulation in the metabolic pathways of hexosylceramides and PC. We describe, for the first time, that this profile includes a subset of lipids that do not change between CO and centenarians, which can be considered stable biomarkers, and another subset of lipids with different levels between CO and centenarians. We hypothesize that this second subset of lipids changes with aging and provides a more favorable profile in CO than in NCO.

### 4.1 Extreme longevity genotype is associated with a healthier biological status

Our results showed that extreme longevity genotype (both centenarians and CO) has better profiles based on fasting glucose levels and BMI. A healthier biological status has been previously described in centenarians (Cruces‐Salguero et al., 2023) and their offsprings (Belenguer‐Varea et al., 2023). In contrast to transcriptomic studies (Inglés et al., 2022), the global lipidome profile analyses showed more restrictive changes related to extreme longevity genotype. In this sense, aging itself has more impact on the lipidome than extreme longevity genotype. The key difference may lay in our focus on the final stage of the omic cascade, where age could affect specific metabolic pathways, especially those related to lipid metabolism (Sol et al., 2023).

### 4.2 Extreme longevity genotype is associated with a phenotypic upregulation of glycosphingolipids

**FIGURE 5:**
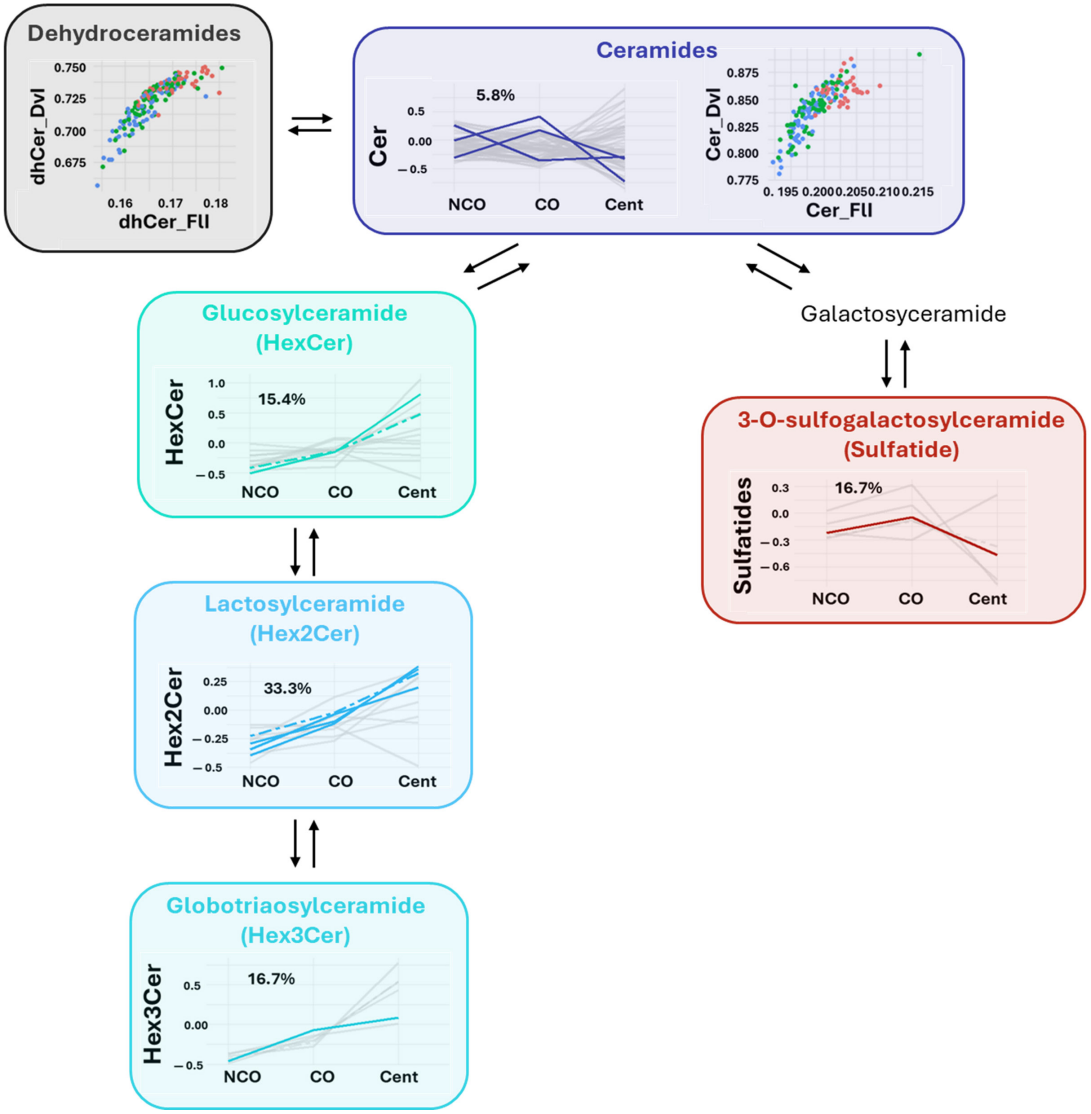
Hexosylceramide formation is favored in extreme longevity. FlI is calculated according to chain length and double bonds of their fatty acids. DvI represents the probability that two lipids randomly selected from a sample will belong to different species. Higher diversity values indicate higher diversity of lipids within the specified lipid class. Cent, Centenarians; Cer, Ceramide; CO, Centenarians' offspring; dhCer, Dihydroceramide; DvI, Diversity Index; FlI, Fluidity Index; Hex2Cer, Hexosyl2ceramides; Hex3Cer, Hexosyl3ceramides; HexCer, Hexosylceramides; NCO, Noncentenarians' offspring.

According to Quehenberger et al. (Quehenberger et al., 2010), plasma sphingolipids account for 5% of total lipids (96% sphingomyelins and 3% ceramides). In addition, it has been estimated that the percentage of hexocylceramides and hexocyl2ceramides of total lipid content are 0.07 and 0.02%, respectively (Chew et al., 2019). Our data are aligned with these studies: sphingolipids represent the 11.5% of all the lipidome detected while the glycosphingolipids the 0.26% (hexocylceramides the 0.16% and hexocyl2ceramides the 0.07%). So, sphingolipids are present at a low content in plasma. Interestingly, small amounts of sphingolipids have also been observed at the tissue level, although their relative concentration varies depending on the tissue (Pradas et al., 2018). Despite being present at relatively low levels compared to other lipids, sphingolipids remain one of the primary lipid categories in all eukaryotic cells. In fact, they are a highly conserved component of cell membranes, inherently predisposed to forming lipid rafts (Posse de Chaves & Sipione, 2010), responsible for a wide range of essential cellular functions, such as: (i) regulating membrane properties like fluidity, organization, protein interactions, trafficking, and signal transduction, and (ii) contributing to key biological processes such as cell identity, oxidative stress, apoptosis, survival, autophagy, cell cycle, proliferation, migration, and senescence. Furthermore, they are also implicated in several pathological conditions (Hannun & Obeid, 2018; Meikle & Summers, 2017; Rietveld & Simons, 1998; Trayssac et al., 2018) and defining mean and maximum longevity (Guillas et al., 2001; Huang et al., 2013; Rao et al., 2007; Schorling et al., 2001; Yang et al., 2010). So, sphingolipid metabolism has been extensively related to aging and age‐related diseases (Berkowitz et al., 2022; Dehghan et al., 2022; Li & Kim, 2021; McGrath et al., 2020). However, little information about the levels and regulation of hexosylceramides, a subclass of glycosphingolipids, is available. In this work, we demonstrated that extreme longevity genotype implies different regulation of the ceramide pathway (Figure 2 and Figure 5). Our findings indicate that the extreme longevity genotype promotes the formation of hexosylceramides from ceramides, concomitantly reducing both the content of specific ceramides and sulfatides. In line with this, previous studies demonstrated that increased levels of ceramides, dihydroceramides, sphingomyelins, and sulfatides and decreased levels of glycosphingolipids are related to accelerating aging and age‐related diseases (Berkowitz et al., 2022; Couttas et al., 2018; Liu et al., 2023). In contrast, higher levels of hexosylceramides and, specifically, lactosylceramides have been previously related to healthy aging and cognitive performance (Barbacini et al., 2022; Dehghan et al., 2022; Liu et al., 2023; Pradas et al., 2022). The results suggest that the enrichment of ceramides during aging (due to the increased de novo synthesis of these sphingolipids) are more strictly regulated in centenarians and their offsprings, favoring the conversion of ceramides to glycosylceramides (Barbacini et al., 2022). This protective mechanism is already active in CO around the age of 70 and becomes even more activated with advancing age, suggesting an intrinsic adaptive program of this pathway in the extreme longevity genotype. In line with this, glycosphingolipids play a key role in determining cellular identity (Russo et al., 2018). A loss of cellular identity has been described during the aging process (Izgi et al., 2022). In this context, we propose that the phenotypic enrichment in glycosphingolipids that we have observed in the long‐lived genotype could be a key component for better maintenance of cellular identity, which would favor extreme longevity.

Our observations also demonstrated that the diversity and fluidity of dehydroceramides and ceramides are increased in extreme longevity, indicating that not only the amount of specific lipid species is important to determining longevity but also the composition of the sphingolipidome. The potential implications of these structural changes in these particular lipid species need to be elucidated, but we hypothesize that these changes in composition have a direct impact on cell functionality. Specifically, it has been previously demonstrated that ceramides, even being a minor constituent of cellular membranes, have a great impact on lipid raft structure (Kinoshita & Matsumori, 2022) and, when incorporated into cellular membranes, confer specific properties not shared by almost any other membrane lipid (Alonso & Goñi, 2018). Among these properties, it is worth noting that they increase the rigidity of the phospholipids in the membranes and make the lipid bilayers and membranes more permeable to solutes. In addition, the presence of dehydroceramides significantly contributes to these two properties (Alonso & Goñi, 2018). Although there is no literature available that relates the physicochemical properties of the ceramides and dehydroceramides and their physiological and pathological function, the changes in the composition of the total pool of these lipid species could be crucial to adapt the membranes to aging and confer a protective environment to achieve successful aging. Interestingly, the diversity of these lipid classes increased in extreme longevity genotypes. CO presented higher diversity of ceramides and dehydroceramides than NCO, and the diversity in centenarians is even higher, suggesting that the promotion of successful aging requires higher diversity to better adapt to aging.

### 4.3 Extreme longevity genotype is associated with an upregulation of ether‐linked PC

**FIGURE 6:**
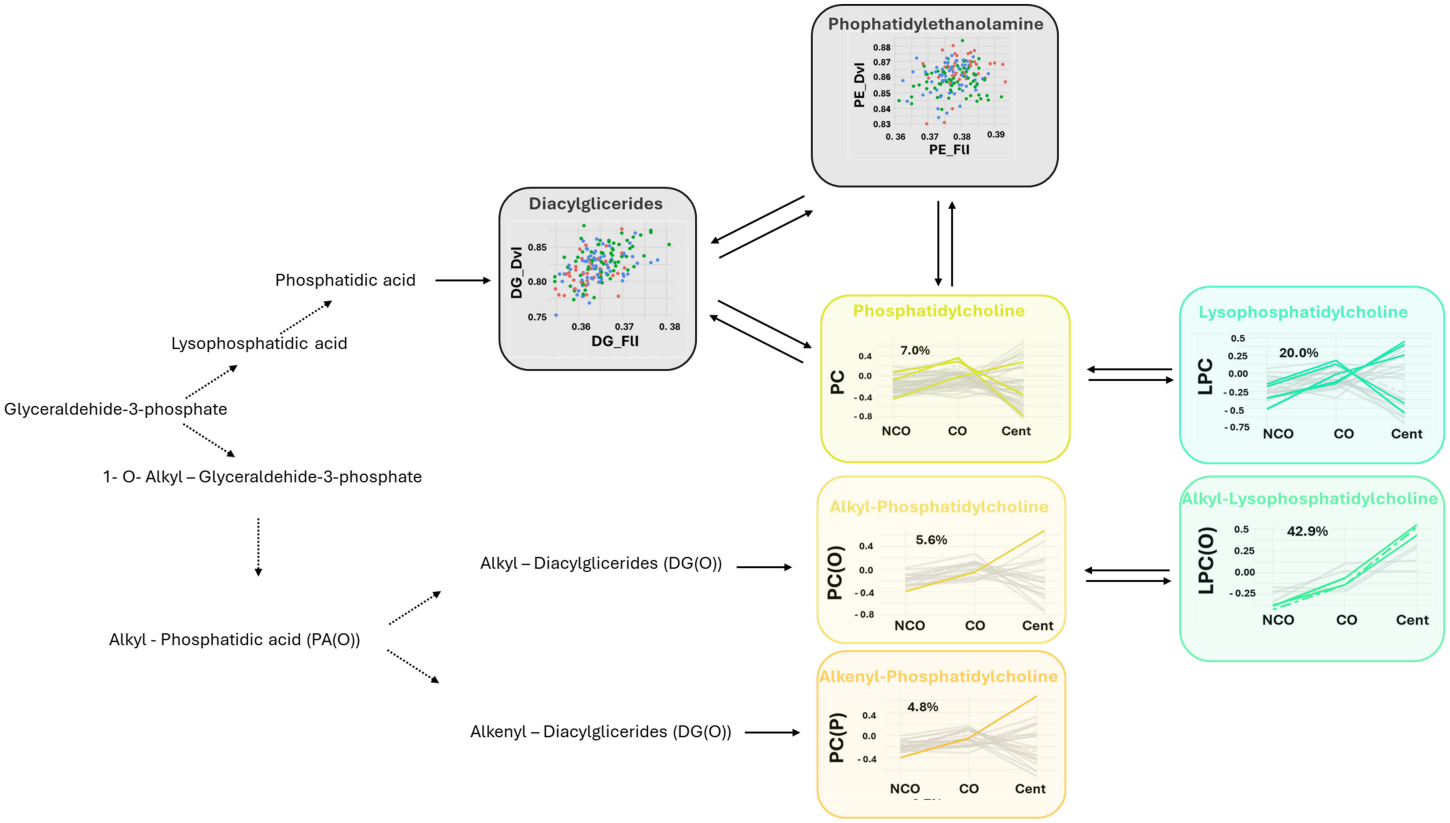
Ether‐linked phosphatidylcholines are increased in extreme longevity. FlI is calculated according to chain length and double bonds of their fatty acids. DvI represents the probability that two lipids randomly selected from a sample will belong to different species. Higher diversity values indicate higher diversity of lipids within the specified lipid class. Cent, Centenarians; CO, Centenarians' offspring; DG, Diglyceride; DvI, Diversity Index; FlI, Fluidity Index; LPC(O), Lysophosphatidylcholine(O); LPC, Lysophosphatidylcholine; NCO, Noncentenarians' offspring; PC(O), Phosphatidylcholine(O); PC(P), Phosphatidylcholine(P); PC, Phosphatidylcholine; PE, Phosphatidylethanolamine.

Phospholipids are the main compounds of cellular membranes and regulate lifespan and healthspan through different cellular and molecular mechanisms (Dai et al., 2021). Globally, phospholipid content (including PC, PE, and phosphatidylserine) decreases with age, but the mechanisms through which different phospholipids contribute to aging and longevity can vary greatly. These mechanisms include changes in membrane fluidity, alteration of the autophagy process, or regulation of redox metabolism (Dai et al., 2021). Our work suggested a global adaptation of PC metabolism in extreme longevity with a special contribution of ether lipids (Figure 2 and Figure 6).

Ether lipid metabolism has been previously related to aging and longevity (Cedillo et al., 2023; Pradas et al., 2019). Ether lipids have structural roles in the cell membrane by promoting tighter packing and reducing fluidity and are in high concentration in lipid raft microdomains. They facilitate membrane trafficking and are crucial in cell signaling, interacting with key pathways and serving as precursors for important signaling molecules (Jové et al., 2023). Furthermore, the presence of the ether bond confers their antioxidant properties (Maeba et al., 2007; Pradas et al., 2019).

A specific PC signature of centenarians has been previously suggested (Collino et al., 2013; Gonzalez‐Covarrubias et al., 2013; Montoliu et al., 2014). This signature implies a global increase of PC in centenarians. Our results show an enrichment of specific PC in centenarians and their offspring, becoming much more evident when we focus on ether‐linked PC. These ether‐PC are increased in CO when compared to NCO and are even more increased in centenarians, suggesting an adaptive role of these species to achieve extreme longevity. When we focused on specific lipid species, we found that 3 PC, 5 LPC, 1 ether‐linked PC, and 2 ether‐linked LPC are increased in extreme longevity phenotype (CO and/or centenarians). These results are especially important for LPC because these lipids are overrepresented (20% of total LPC detected are different and 42.9% of LPC(O)). Furthermore, total ether‐linked LPC is increased in extreme longevity genotypes (CO and centenarians). These results are in line with previously published papers where the role of PC and LPC in extreme longevity has been explored (Gonzalez‐Covarrubias, 2013; Gonzalez‐Covarrubias et al., 2013; Liu et al., 2023; Pradas et al., 2019; Semba et al., 2019). Globally, the general decrease of ether lipids observed in aged individuals (Maeba et al., 2007) is reversed in the extreme longevity genotype (CO and centenarians), as an adaptive response to aging process. This particular signature could be protective from oxidative stress and inflammation.

The diversity of phospholipids, which differ largely in the lengths of the chains and the number and position of the double bonds, may affect the cellular signaling properties of the membranes. The fluidity of the membrane, which depends on both fatty acyl chain length and number of double bonds, regulates numerous signaling pathways and cellular processes that are crucial for human health and disease (Dai et al., 2021). Furthermore, membrane fluidity is decreased with the incorporation of cholesterol (Chakraborty et al., 2020). In line with this, the fact that we did not find statistically significant differences in free cholesterol levels suggests that the physicochemical changes in cell membranes in extreme longevity should be attributed to other classes of lipids, such as sphingolipids or phospholipids. In the present work, we observed different DvI of DG and PE in extreme longevity genotypes. Specifically, DG diversity presents lower levels in centenarians, having the CO at an intermediate level. Contrarily, a higher diversity of PE species is found in centenarians. These results suggest a lipid class‐specific adaptive response in terms of diversity to achieve extreme longevity.

Based on our findings, several questions remain unresolved and should be addressed in future research: (i) given the unique lipidomic composition of each tissue in the body, our understanding remains limited regarding how different tissues contribute to the observed plasma lipidomic profile, (ii) because we analyzed the plasma global lipidome, the changes observed in lipid species could not be ascribed to a specific lipoprotein or to their metabolism; (iii) although it is previously demonstrated that there is an exchange between lipoprotein components and cell membranes (Waldie et al., 2020), we do not know how plasma lipid species could specifically affect tissular lipidome, and (iv) although we analyzed more than 500 lipid species, covering all described lipid classes, it is important to note that a portion of the lipidome is not included in the present study. In fact, there is no single analytical platform that can capture the entire lipidome due to the vast diversity of compound and their physicochemical properties.

## 5 CONCLUSIONS

In this work, we characterized, for the first time, the plasma lipidome of centenarians and CO to provide a lipidomic signature of extreme longevity. The extreme longevity lipidomic signature is characterized by (i) a global enrichment of hexosylceramides, (ii) a decrease of specific species of ceramides and sulfatides, (iii) a global increase of ether‐PC and ether‐LPC and (iv) changes in the fluidity and diversity of specific lipid classes. We propose that this signature is the result of an adaptive response to aging, and we point out the conversion of ceramides to hexosylceramides and the maintenance of the levels of the ether‐linked PC as phenotypic trait to guarantee extreme longevity.

## AUTHOR CONTRIBUTIONS

Conceptualization, J.V., C.B., M.J., and R.P.; methodology, and formal analysis, all authors; statistical analysis, J.S., M.J., and R.P.; results discussion, interpretation, and writing—original draft preparation, all authors; writing—review and editing, C.B., M.J., and R.P.; study supervision, C.B., M.J., and R.P; funding acquisition, J.V., C.B., M.J., and R.P. All authors have read and agreed upon the published version of the manuscript. C.B., M.J., and R.P. are the guarantors of this work, and, as such, had full access to all the data in the study and take responsibility for the integrity of the data.

## FUNDING INFORMATION

This research was funded by the Spanish Ministry of Science, Innovation, and Universities (grant PID2023‐152233OB‐I00, cofunded by the European Regional Development Fund, “A way to build Europe”), the Diputació de Lleida (PP10605‐PIRS2021), and the Generalitat of Catalonia: Agency for Management of University and Research Grants (2021SGR00990) to R.P. This work was also supported by Instituto de Salud Carlos III (PI24/01431) and Diputació de Lleida (PP10845‐PIRS2023) to MJ. This work was also supported by the following grants: Instituto de Salud Carlos III CB16/10/00435 (CIBERFES), EU Funded H2020‐DIABFRAIL‐LATAM (Ref: 825546), Instituto de Salud Carlos III (ISCIII) (Ref. AC20/00026) under the umbrella of the European Joint Programming Initiative ‘A Healthy Diet for a Healthy Life’ (JPI HDHL) and the ERA‐NET Cofund ERA‐HDHL (GA N° 696295 of the EU Horizon 2020 Research and Innovation Programme) to JV. Grants PID2020‐113839RB‐I00 funded by Spanish Ministry of Science, Innovation, and Universities (MCIN/AEI/10.13039/501100011033) and CIAICO/2022/190 funded by Conselleria de Educació, Universitats y Ocupació to CB. IRBLleida is a CERCA Programme/Generalitat of Catalonia.

## CONFLICT OF INTEREST STATEMENT

The authors declare that this research was conducted in the absence of any commercial or financial relationship that could be considered as a potential conflict of interest.

## INCLUSION AND DIVERSITY

For studies involving human subjects, whether recruited (e.g., clinical analyses) or enrolled spontaneously (e.g., online surveys), we worked to ensure sex balance in the recruitment of human subjects. We worked to ensure ethnic or other types of diversity in the recruitment of human subjects. We worked to ensure that the study questionnaires were prepared in an inclusive way.

## INSTITUTIONAL REVIEW BOARD STATEMENT

The study was conducted according to the guidelines of the Declaration of Helsinki, the guidelines of Spanish legislation (Real Decreto 1716/2011), and the approval of the local ethics committees.

## Supporting information


Data S1.

Figure S1.

Table S1.

## Data Availability

The lipidomics dataset used for the analyses is available in https://dataverse.csuc.cat/dataset.xhtml?persistentId=doi:10.34810/data1748. Doi: https://doi.org/10.34810/data1748.
