# Supplementary figures and images for: Phenotypic upregulation of hexocylceramides and ether‐linked phosphocholines as markers of human extreme longevity
Source: Aging Cell. 2024 Dec 5;24(4):e14429. doi: 10.1111/acel.14429 (PMC11984674; doi:10.1111/acel.14429)

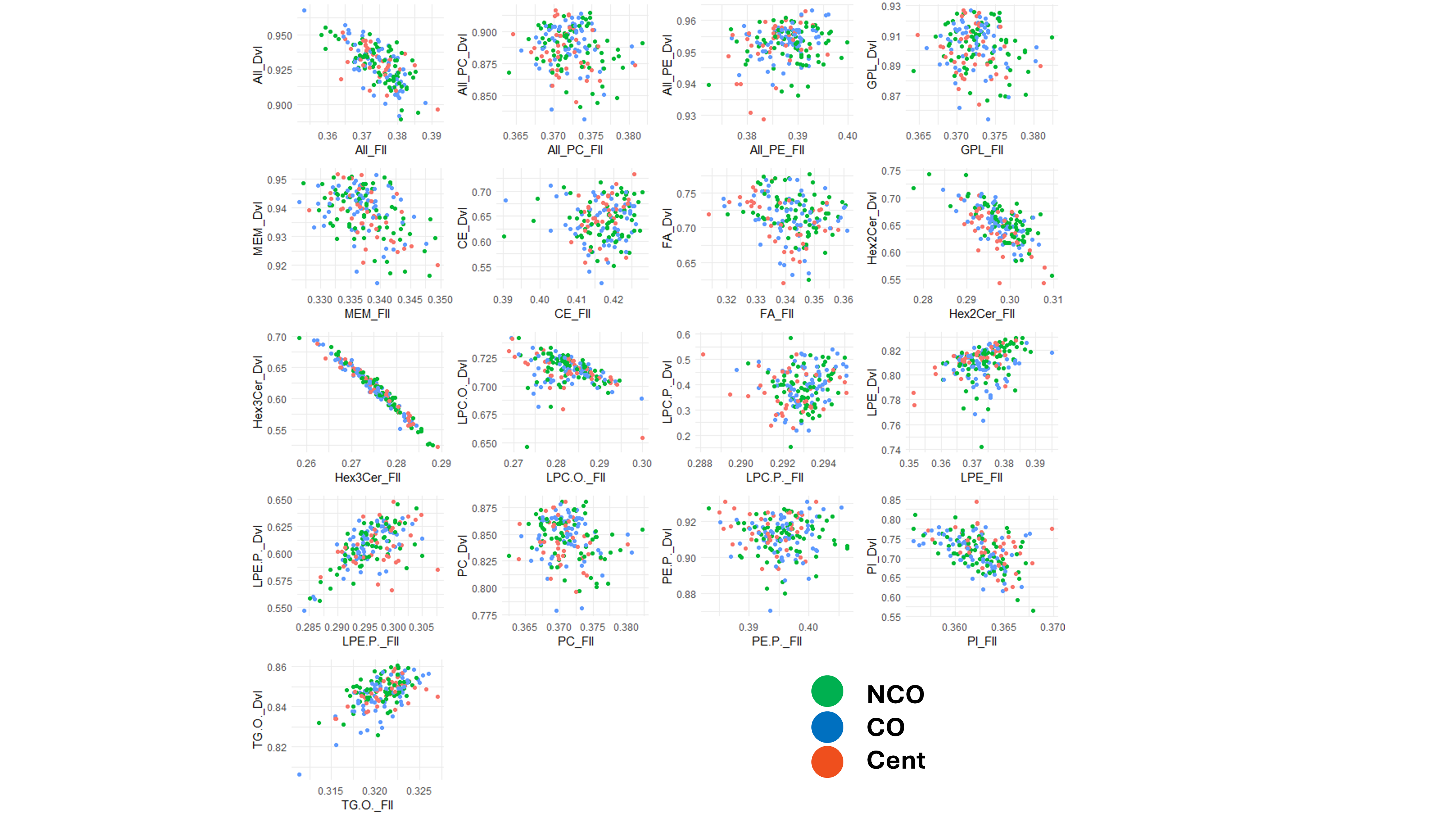

Supplement: Supplementary file 2 — Figure S1. [file ACEL-24-e14429-s003.tif]
