# Supplementary material for: Phenotypic upregulation of hexocylceramides and ether‐linked phosphocholines as markers of human extreme longevity
Source: Aging Cell. 2024 Dec 5;24(4):e14429. doi: 10.1111/acel.14429 (PMC11984674; doi:10.1111/acel.14429)
Supplement: Supplementary file 3 — Table S1. [file ACEL-24-e14429-s002.docx]

**Supplementary Table 1.** Class-representative internal lipid standards.

| **ISTD** | | **Reference** | |
| --- | --- | --- | --- |
| LPI 17:0 | 17:0 Lyso PI-d5 | 850108 | Avanti |
| dhCer(d18:0/13:0 d7) | C13-dihydroceramide-d7(d18:0-d7/13:0) | 330726P | Avanti |
| HexCer(d18:1/15:0) d7 | C15 Glucosyl(β) Ceramide-d7 (d18:1-d7/15:0) | 330729P | Avanti |
| Hex2Cer(d18:1/15:0) d7 | C15 Lactosyl(β) Ceramide-d7 (d18:1-d7/15:0) | 330727P | Avanti |
| Hex3Cer(d18:1/17:0) | N-Heptadecanoyl-ceramide trihexoside | 1523 | Matreya LLC |
| 16:0-d31-18:1 PA | 16:0-d31-18:1 Phosphatidic acid | 860453C | Avanti |
| Sph(d17:1) | Sphingosine (d17:1) | 860640P | Avanti |
| Sulfatide (d18:1:/24:1 d7 ) | C24:1 mono-sulfo galactosyl(ß) ceramide-d7 (d18:1/24:1) | 860736E | Avanti |
| AcylCarnitine(18:1 d9) | C18:1 L-carnitine-d9 | 870321P | Avanti |
| Cer(d18:1/17:0) | C17 Ceramide (d18:1/17:0) | 860517P | Avanti |
| LPC(13:0) | 13:0 Lyso PC | 855476P | Avanti |
| LPE 18:1 d7 | 18:1-d7 Lyso PE | 791644C | Avanti |
| PC(13:0) | Phosphatidylcholine 13:0 | 850340P | Avanti |
| PE(17:0) | Phosphatidylethanolamine 17:0 | 830756P | Avanti |
| PG(17:0) | Phosphatidylglycerol 17:0 | 830456P | Avanti |
| PS(17:0) | Phosphatidylserine 17:0 | 840028P | Avanti |
| TG(17:0/17:0/17:0) | Triglyceride 51:0 | T2151 | Sigma |
| FA(22:6) d5 | Fatty acid 22:6 d5 | 733326 | Sigma |
| DG 16:1 d5 | 1,3-16:1 D5 DG | 800853C | Avanti |
| SM (18:1/18:1) d9 | 18:1 SM (d18:1/18:1)-d9 | 860740L | Avanti |
| CE(18:1) d7 | Cholesterol Ester 18:1 d7 | 700185M | Avanti |
| COH d7 | cholesterol-d7 | 700041P | Avanti |
| ox-18:2 CHO linoleate d7 | ox-18:2 cholesteryl linoleate (d7) | 700191P | Avanti |
| 8-isoPF2a d4 | 8-iso Prostaglandin F2a d4 | 316350 | Cayman |
| 7b OH CHO d7 | 7-hydroxy cholesterol d7 | 25548 | cayman |
